# Multi-Mycotoxin Contamination in Serbian Maize During 2021–2023: Climatic Influences and Implications for Food and Feed Safety

**DOI:** 10.3390/toxins17050227

**Published:** 2025-05-04

**Authors:** Felipe Penagos-Tabares, Anastasija Todorov, Jog Raj, Hunor Farkaš, Goran Grubješić, Zdenka Jakovčević, Svetlana Ćujić, Jelena Nedeljković-Trailović, Marko Vasiljević

**Affiliations:** 1 CIBAV Research Group, Veterinary Medicine School, Faculty of Agrarian Sciences, Universidad de Antioquia, 050034 Medellín, Colombia; 2 Agromed Austria GmbH, 4550 Kremsmünster, Austria; grubjesic@agromed.at; 3 Department of Animal Nutrition and Botany, Faculty of Veterinary Medicine, University of Belgrade, Bulevar Oslobodjenja 18, 11000 Belgrade, Serbiatjelena@vet.bg.ac.rs (J.N.-T.); 4 PATENT CO., 24211 Mišićevo, Serbia; jog.raj@patent-co.com (J.R.); hunor.farkas@patent-co.com (H.F.); zdenka.jakovcevic@patent-co.com (Z.J.); svetlana.cujic@patent-co.com (S.Ć.); marko.vasiljevic@patent-co.com (M.V.)

**Keywords:** mycotoxins, maize, climate, food safety, fumonisins, aflatoxins, Serbia, co-contamination

## Abstract

Mycotoxin contamination in maize poses significant food and feed safety risks, particularly in regions with variable climatic conditions like Serbia. This study investigated the occurrence of regulated mycotoxins in maize harvested across the Republic of Serbia from 2021 to 2023, emphasizing the impact of climatic factors. A total of 548 samples of unprocessed maize grains were analysed for the presence of key mycotoxins, including aflatoxins, ochratoxin A, zearalenone, deoxynivalenol, fumonisins, and trichothecenes type A (T-2 and HT-2 toxins), using validated analytical methods. The results revealed high contamination frequencies, with aflatoxins and fumonisins being the most prevalent. The results revealed substantial temporal variability and frequent co-contamination of mycotoxins. Aflatoxin B_1_ (AFB_1_) was the most concerning contaminant, with 73.2% of the samples in 2022 exceeding the European regulatory limit for human consumption (5 µg/kg) for un processed maize grains, reaching peak concentrations of 527 µg/kg, which is 105.4 times higher than the allowed limit. For animal feed, the limit of 20 µg/kg was exceeded in 40.5% of the samples, with the highest concentration being 26.4 times greater than the maximum allowable level. In 2021, the non-compliance rates for AFB_1_ in food and feed were 8.3% and 2.3%, respectively, while in 2023, they were 23.2% and 12.2%, respectively. Fumonisins contamination was also high, particularly in 2021, with fumonisin B_1_ (FB_1_) detected in 87.1% of samples and average concentrations reaching 4532 µg/kg. Although levels decreased in 2023 (70.7% occurrence, average 885 µg/kg), contamination remained significant. Deoxynivalenol (DON) contamination was consistently high (>70% of samples), with peak concentrations of 606 µg/kg recorded in 2021. Zearalenone (ZEN) and ochratoxin A (OTA) occurred less frequently, but ZEN levels peaked in 2022 at 357.6 µg/kg, which is above the regulatory limit of 350 µg/kg for food. Trichothecenes (HT-2 and T-2 toxins) were detected sporadically, with concentrations well below critical thresholds. Co-occurrence of mycotoxins was frequent, with significant mixtures detected, particularly between aflatoxins and fumonisins, as well as other fusarial toxins. The analysis demonstrated that temperature, humidity, and rainfall during both the growing and harvest seasons strongly influenced mycotoxin levels, with the most severe contamination occurring under specific climatic conditions. Notably, the highest mycotoxin levels, like aflatoxins, were linked to warmer temperatures and lower rainfall. The high non-compliance rates for aflatoxins and fumonisins and co-contamination pose significant food and feed safety risks. From a public health perspective, chronic exposure to contaminated maize increases the likelihood of carcinogenesis and reproductive disorders. Reduced productivity and bioaccumulation in animal tissues/products represent serious economic and safety concerns for livestock. This study provides insights into the potential risks to food and feed safety and the need for enhanced regulatory frameworks, continuous monitoring, and mitigation strategies in Serbia as well as other geographical regions.

## 1. Introduction

Maize (*Zea mays* L.), also known as corn, is one of the most important cereal crops globally, essential for human and animal nutrition [1,2]. In Serbia, maize is crucial in agricultural production, accounting for a significant portion of the country’s arable land. The favourable climatic conditions of the region, linked with Serbia’s rich agrarian culture, make it one of the leading producers and exporters of maize in Southeast Europe [3,4]. Maize represents a key component in Serbia’s domestic consumption and export economy, with its derivatives widely used in food, feed, and industrial applications. Its relevance extends beyond its economic value, as maize plays a fundamental role in the Serbian diet, particularly in traditional dishes, processed food products, and livestock feed exports. In the Republic of Serbia, around 80% of the maize is used for animal feeding, while humans and the food industry mainly consume the remaining amount. The use of maize in Serbia and its production and the factors affecting the production of mycotoxins in maize have been expressed in detail elsewhere [5]. In 2022, Serbia was ranked as the world’s 15th largest exporter of maize, and this crop was the 9th most exported product. The main destinations of maize exports from Serbia were Romania, South Korea, Italy, Bosnia and Herzegovina, and Hungary [6,7].

However, maize’s importance in the food and feed sectors is associated with concerns over contamination by mycotoxins, toxic secondary metabolites produced by certain species of fungi. Over 400 mycotoxins have been currently identified, but some mycotoxins, such as aflatoxins (AFs: AFB_1_, AFB_2_, AFG_1,_ and AFG_2_), ochratoxin A (OTA), deoxynivalenol (DON), zearalenone (ZEN), T-2 and HT-2 toxins, and fumonisins (Fs: fumonisin B_1_ (FB_1_) and fumonisin B2 (FB_2_), respectively), are contemplated in the European legislation and are frequent contaminants in maize, especially in regions with warm and humid climates [5,8]. These toxins pose a severe threat to food and feed safety due to their potential health impacts related to the teratogenic, nephrotoxic, hepatotoxic, neurotoxic, mutagenic, carcinogenic, and immunosuppressive effects, and they are among the most significant mycotoxins affecting the health of humans and animals [9,10]. In the last 2–3 decades, mycotoxin contamination in feed and food chains has received attention due to new legislative limits on maximum allowable contamination levels in many countries worldwide [11]. Contaminated maize risks the health of humans who consume maize-based products and animals fed with contaminated maize, which can lead to animal health issues and reduced productivity. Additionally, bioaccumulation of toxins in the food chain further intensifies public health threats [12].

Commodities in the food and feed chains are contaminated with multiple mycotoxins, which occur after colonisation by a mixed community of mycotoxigenic fungi. Contamination can occur at different stages of the maize production chain, with distinct differences between field (pre-harvest) and storage (post-harvest) contamination. Field contamination typically occurs during the growing season when environmental factors such as temperature, humidity, and rainfall create favourable conditions for fungal proliferation and toxin production. For example, warm temperatures (25–30 °C) and high humidity (>70%) favour the growth of *Aspergillus* spp., leading to aflatoxin production, while moderate temperatures (18–22 °C) combined with high rainfall favour *Fusarium* spp., resulting in fumonisin and deoxynivalenol (DON) contamination. Agronomic practices, plant stress, and biological interactions further influence field contamination levels. In contrast, storage contamination usually results from inadequate drying and moisture control after harvest, where *Aspergillus* and *Penicillium* spp. can proliferate, producing aflatoxins and ochratoxin A, respectively [5,13,14,15]. Recent surveys of mycotoxin contamination levels in raw commodities (wheat, corn, soybeans) and compound mixed feeds have revealed the ubiquitous presence of mycotoxins [5,13,16,17]. The co-occurrence of mycotoxins creates concerns, as the combined toxic effect may be additive, antagonistic, or synergistic and have consequences for animal husbandry [18,19,20]. Mycotoxin contamination of animal feeds reduces animal growth rates and can have a considerable spectrum of toxic effects on different groups of animals [10,21].

Mycotoxin contamination in maize is particularly concerning in Serbia, given the country’s climatic variability, which influences fungal growth and mycotoxin production [22]. With extreme weather events becoming more frequent due to climate change, the risk of mycotoxin contamination has heightened as fungi thrive in environments characterised by fluctuating temperatures and humidity levels [23,24,25]. Consequently, monitoring and controlling mycotoxin levels in maize is crucial for safeguarding public health, ensuring food and feed safety, and maintaining the quality of agricultural exports [26]. These have shown that weather/changing climate conditions in different regions have influenced the trends in the predominant mycotoxins present in diverse types of feed/food crops [22]. Mycotoxin contamination in maize from Serbia has been previously reported, with a high presence of AFs [27] and DON [28]. These reports indicate that the human population and livestock have been exposed to a high risk of specific mycotoxins in recent years. Recently, Serbia has experienced climate changes that have affected corn’s mycotoxins [22,28,29]. Thus, this study aims to investigate the co-occurrence and levels of regulated mycotoxins (AFB_1_, AFB_2_, AFG_1_, AFG_2_, OTA, ZEN, DON, FB_1_, FB_2_, T-2, and HT-2) in Serbian maize harvested from 2021 to 2023. The trends in the co-occurrence of different mycotoxins in maize over three years (2021–2023) were compared and described in detail. The influence of geo-climatic factors such as temperature, humidity, rainfall, and altitude on the mycotoxin contamination levels was also explored.

## 2. Results

### 2.1. Climatic Conditions in Serbia During the Maize Growing and Harvest Seasons of 2021 to 2023

The climatic conditions across Serbian maize-growing regions varied significantly from 2021 to 2023. Table 1 details the geo-climatic factors, including temperature, humidity, and rainfall, during the growing (June–September) and harvest (September) periods. Average growing season temperatures remained stable (21.2 °C in 2021–2022, 21.5 °C in 2023), while harvest temperatures fluctuated, with 2023 being the warmest (19.8 °C vs. 16.9 °C in 2021 and 16.2 °C in 2022). Relative humidity rose during the growing season (62.2% in 2021 to 70.5% in 2023) but declined during harvest (76.3% in 2021 to 70.8% in 2023). Rainfall varied more, peaking in 2022 (305.5 mm) and dropping during the harvest months, especially in 2022 (11.6 mm) and 2023 (13.5 mm) compared to 2021 (70.2 mm). The study locations ranged in altitude from 42 to 2017 m.a.s.l.

### 2.2. Occurrence and Concentration of Detected Regulated Mycotoxins

The occurrence and contamination levels of regulated mycotoxins in Serbian maize harvested between 2021 and 2023 varied significantly across the years (Table 2). Contamination with AFs, particularly AFB_1_, showed a marked increase in 2022, with an occurrence of 73.2% and an average concentration of 80.6 ± 115.3 µg/kg. This was significantly higher than in 2021 (18.5% occurrence; 10 ± 16.5 µg/kg) and 2023 (34.1% occurrence; 29.2 ± 43.3 µg/kg). While AFG_1_ and AFG_2_ were detected sporadically, their presence was rare compared to AFB_1_ and AFB_2_, with no detection of AFG_1_ and AFG_2_ in 2023. No instances of contamination with AFB_2_, AFG_1_, or AFG_2_ without the presence of AFB_1_ were observed. Therefore, the occurrence rate of the sum of AFs aligns with the occurrence rate of AFB_1_. Concerning *Fusarium*-produced mycotoxins, their occurrence was >70%, with the highest record in 2021 (89.4%). FB_1_ and FB_2_ were consistently prevalent over the three years. In 2021, 87.1% of samples contained FB_1_, with contamination levels reaching 4532 ± 4617 µg/kg. Although FB levels decreased in 2023 (70.7% occurrence; 885 ± 981 µg/kg), they still represented a significant contamination risk, especially given their synergy with AFs in maize-based products. DON was also frequently detected, with an occurrence of over 70%. In 2021, DON contamination was recorded in 87.5% of samples, with an average concentration of 291 ± 188 µg/kg. Although DON levels slightly decreased in 2023 (222 ± 123 µg/kg), contamination remained significant. ZEN was detected at a lower occurrence rate (<4% during the three years), with peak contamination in 2022 (357.6 ± 347 µg/kg). Like ZEN, HT-2 and T-2 toxins were less frequent than the other mycotoxins, with levels remaining below critical thresholds in most years. However, in 2023, HT-2 contamination reached an average of 84.3 ± 31.7 µg/kg in 1.2% of the samples, suggesting a year-to-year variation potentially driven by environmental factors. OTA contamination was detected in low proportions, with a peak occurrence of 29.8% in 2022, although average levels remained below regulatory limits.

**Figure 1:**
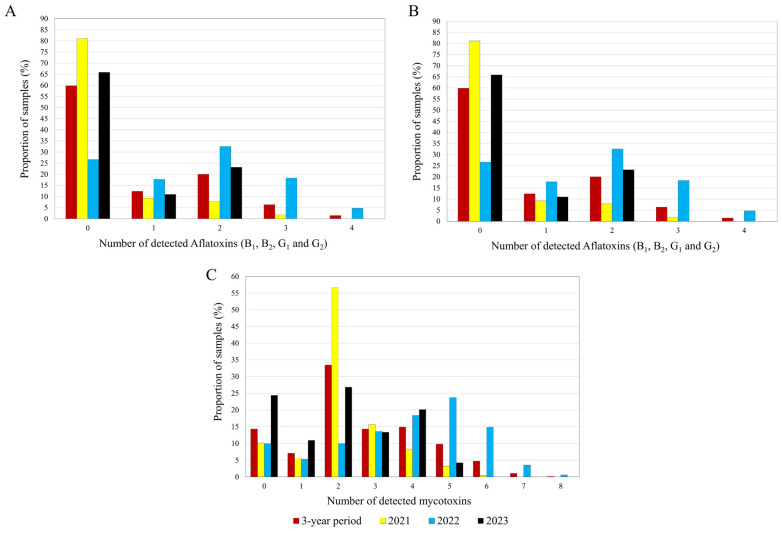
Clustered columns indicating the proportion of samples with co-contamination of (**A**) aflatoxins, (**B**) *Fusarium* mycotoxins, and (**C**) regulated mycotoxins in Serbian maize samples harvested during 2021–2023 (The values are available in Supplementary Table S1).

Table 3 reports the detected combinations of regulated mycotoxins in Serbian maize. During the 3 years, the positive samples contained an average of 3 ± 1 regulated mycotoxins with a maximum of 8 mycotoxins/sample. Co-contamination of maize samples with multiple mycotoxins was a common finding in all years of the study. The average co-contaminating mycotoxins per sample were 2 ± 1, 4 ± 2, and 3 ± 1 for 2021, 2022, and 2023, respectively. During the three years, 27.9% of the analysed maize samples presented two to four AFs. The co-occurrence of AFs was 9.7, 55.6, and 23.2% in 2021, 2022, and 2023, respectively (Figure 1A, Supplementary Data Table S1). During the three-year period, 76.6% of the maize samples presented between two and five regulated *Fusarium*-mycotoxins. Such co-contamination of *Fusarium* mycotoxins ranged from 59.1% (2023) up to 83.4% (2021) (Figure 1B, Supplementary data Table S1). A large proportion of the maize samples (78.5%) were contaminated with two or more mycotoxins during the 3 years, with yearly occurrences of 84.3% (2021), 84.6% (2022), and 64.6% (2023). Figure 1C, Supplementary data Table S1). In 2022, the year with the highest co-contamination rates, primarily aflatoxins and FBs (Table 3). The most frequent combinations involved FB_1_ and FB_2_ (81.9% in 2021 and 79.2% in 2022), followed by AFs (AFB_1_, AFB_2_, AFG_1_, and/or AFG_2_), co-occurring with FBs (FB_1_ and FB_2_) in 69.6% of samples in 2022.

**Figure 2:**
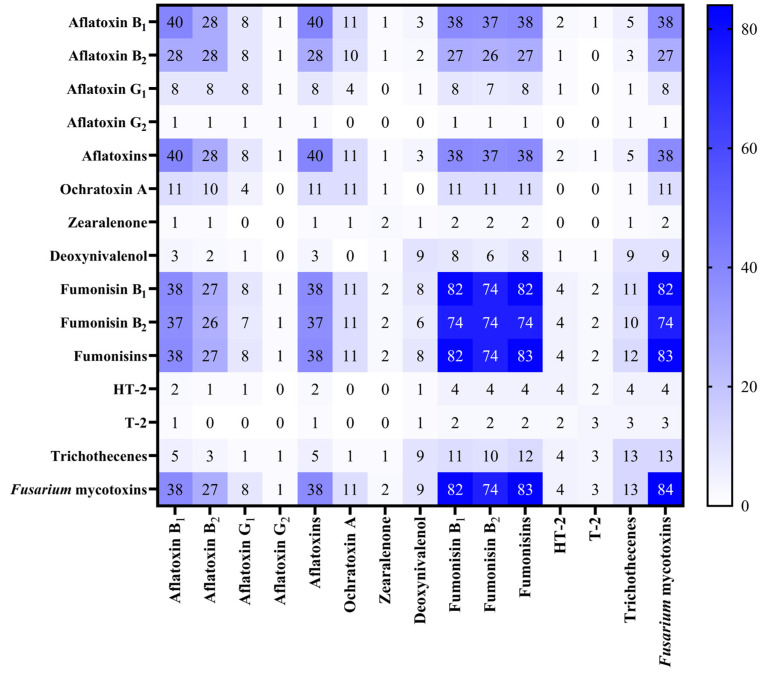
Heatmap indicating the co-occurrence (%) of regulated mycotoxins (and mycotoxin groups) contaminating Serbian maize samples harvested during 2021–2023. Darker blue areas indicate high-risk combinations that warrant particular attention for food and feed safety assessments, while lighter shades suggest less frequent pairings.

The heatmap analysis (Figure 2) indicates the frequency of co-occurrence (%) of regulated mycotoxins (and mycotoxin groups) contaminating Serbian maize samples harvested during 2021–2023. AFs and FBs were present in 38% of the analysed samples (Figure 2). In particular, the 2022 maize harvest presented a high co-contamination rate with these mycotoxins derived from *Fusarium* and *Aspergillus* species, FBs and AFs, respectively (Figure 1A,B, and Table 3). In 2023, although average concentrations of AFs decreased, 29.3% of the samples were still co-contaminated with FBs and AFs. Co-contamination with DON, ZEN, and FBs was less frequent but still detectable, with 1.2% of samples in 2022 and 2023 containing all three toxins (Table 3).

### 2.3. Influences of Climatic Factors on the Concentration of Mycotoxins

**Figure 3:**
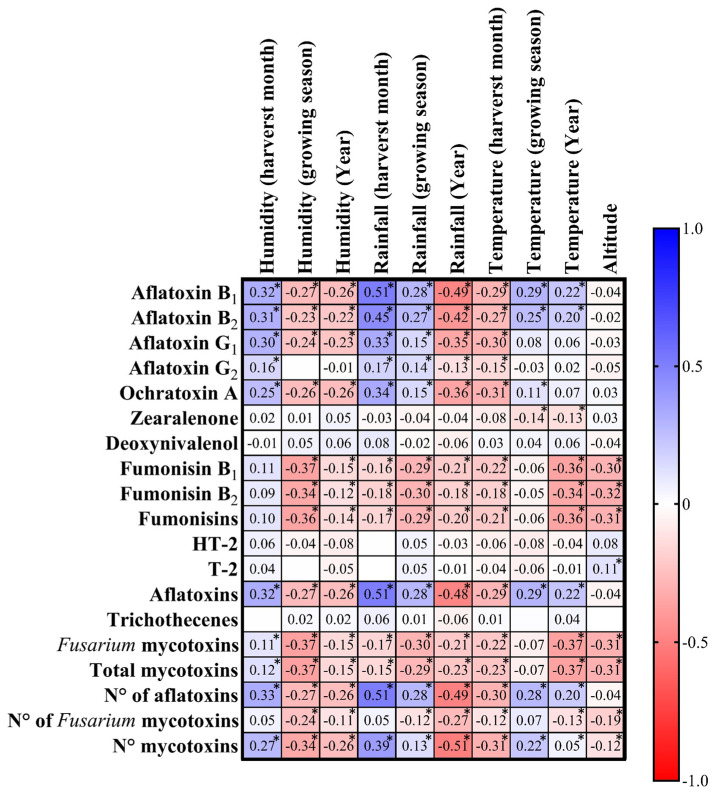
T Heatmap illustration of the Spearman’s correlation coefficients (Rho) among levels of regulated mycotoxins contaminating Serbian corn samples harvested during 2021–2023 and the climatic conditions (humidity, rainfall, temperature, and altitude) of their respective localities of the maize origin. The color gradient from deep blue to deep red represents the strength and direction of correlations, with blue shades indicating positive correlations (Rho values from 0 to +1.0), red shades indicating negative correlations (Rho values from −1.0 to 0), and white areas indicating no correlation (Rho values close to 0). The intensity of the color reflects the magnitude of the correlation, where darker colors indicate stronger relationships. Correlation coefficients marked with an asterisk (*) are statistically significant (*p*-value < 0.01), emphasising robust associations that are unlikely to have occurred by chance. All the *p*-values are available in supplementary data Table S2).

Figure 3 presents a heatmap showing Spearman’s correlation coefficients between regulated mycotoxins (on the left) and climatic factors (on the top) for maize samples collected in Serbia from 2021 to 2023. AFB_1_ and AFB_2_ show a moderate positive correlation with rainfall during the harvest month (Rho = 0.51 for AFB_1_ and AFs, and 0.45 for AFB_2_, *p* < 0.01) and the growing season (Rho = 0.28 for AFB_1_, AFs, and Rho = 0.27 AFB_2_, *p* < 0.01). This indicates that higher rainfall during the harvest month (September) and the maize growing season (June to September) increases AFs contamination. Humidity (growing season) also correlates positively with AFs. These positive moderate correlations were observed mainly between AFs and humidity of the harvest month (Rho ≥ 0.3 for AFB_1_, AFB_2_, AFG_1,_ and AFs, *p* < 0.01), showing that humidity influences the AF levels. The elevated AFs levels in 2022 coincided with higher humidity and rainfall during the growing season. Humidity of the growing season presented a weak negative correlation with AFs levels (Rho = −0.27), suggesting that the roughness during the growing season influences the levels of AFs. The data analysis revealed significant correlations between climatic conditions and mycotoxin contamination levels (Figure 3, Table S2).

**Figure 4:**
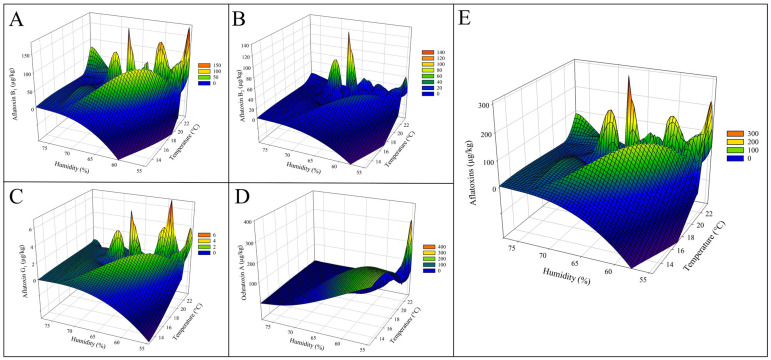
Three-dimensional mesh graphs representing the relationship between the concentrations of aflatoxins, relative humidity (%), and temperature (°C) during the growing season (June to September) in Serbian corn samples harvested between 2021 and 2023. The panels illustrate the effect of climatic factors on aflatoxin concentrations, including (**A**) aflatoxin B_1_ (A, AFB_1_), (**B**) aflatoxin B_2_ (AFB_2_), (**C**) aflatoxin G_1_ (AFG_1_), and (**D**) ochratoxin A (OTA), as well as (**E**) total aflatoxins (sum of AFB_1_, AFB_2_, AFG_1_, and AFG_2_). Higher aflatoxin concentrations are observed under elevated temperature and relative humidity, with prominent peaks highlighting the climatic conditions most conducive to contamination. The color reflects the level of contamination.

**Figure 5:**
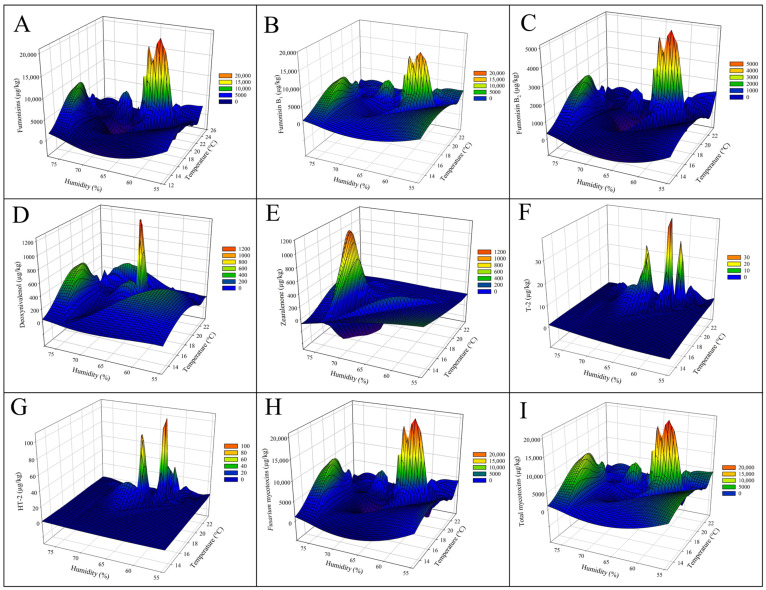
Three-dimensional mesh graphs representing the relationship among the concentrations of various mycotoxins, monthly relative humidity (%), and temperature (°C) during the growing season (June to September) in Serbian corn samples harvested between 2021 and 2023. The graphs illustrate the influence of climatic factors on mycotoxin contamination levels in maize, with warmer and humid conditions often associated with higher concentrations. The panels represent (**A**) fumonisins (FB_1_ + FB_2_), (**B**) fumonisin B_1_ (FB_1_), (**C**) fumonisin B_2_, (**D**) deoxynivalenol (DON), (**E**) zearalenone (ZEN), (**F**) T-2 toxin, (**G**) HT-2 toxin, (**H**) *Fusarium* mycotoxins (Sum of DON, ZEA, FB_1_, and FB_2_), and (**I**) total mycotoxins (Sum of all detected mycotoxins). The color reflects the level of contamination.

**Figure 6:**
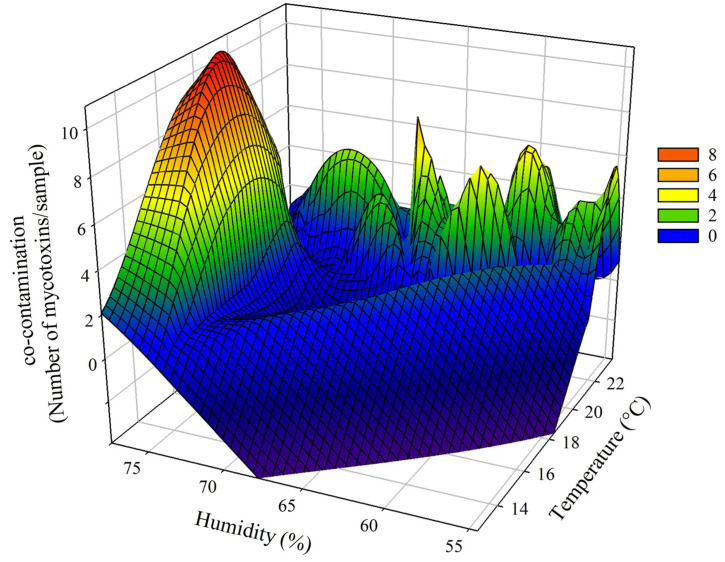
Three-dimensional mesh graph representing the relationship between co-contamination (number of mycotoxins per sample), relative humidity (%), and temperature (°C) during the growing season (June to September) in Serbian corn samples harvested between 2021 and 2023. The graph illustrates how variations in climatic factors influence the degree of mycotoxin co-contamination, with higher levels of co-contamination observed under increased humidity and elevated temperatures. Peaks in the graph indicate the conditions most favourable for multi-mycotoxin contamination detected in the analysed samples. The color reflects the co-contamination level.

The relationship between climatic factors and mycotoxin contamination in Serbian maize samples collected from 2021 to 2023 reveals critical insights into how temperature and relative humidity influenced fungal proliferation and toxin production. An additional representation of the data via 3D mesh graphs, which considered the relationship of temperature and humidity of the growing sections regarding the mycotoxin levels (Figure 4, Figure 5 and Figure 6), showed clearly that higher temperatures promoted, generally, the higher peaks of diverse mycotoxins. Specifically, 2022, which experienced higher-than-average rainfall (305.5 ± 87.3 mm) and humidity (64.4 ± 5.4%) during the growing season, had the highest levels of AFs in maize (Table 1 and Table 2; Figure 5A–C,E). This suggests that warm and moist conditions create a favourable environment for *Aspergillus* species, leading to a surge in AF contamination. Conversely, drier conditions during the harvest period of 2023 may have contributed to slightly lower contamination levels that year.

Aflatoxin concentrations, including AFB_1_, AFB_2_, AFG_1_, AFG_2_, and total aflatoxins, were examined in relation to climatic parameters during the growing season (June to September). The concentrations of AFB_1_ peaked at temperatures between 20 °C and 22 °C and relative humidity levels under 70% (Figure 4A). AFB_2_ followed a similar trend, with its highest concentrations also occurring under warm and non-excessive humid conditions (<70% humidity), although at generally lower levels compared to AFB_1_ (Figure 4B). AFG_1_ and AFG_2_ concentrations showed sporadic peaks under specific climatic conditions of high humidity (>65%) and moderate temperatures; however, the main levels trended towards lower humidity, and these toxins were less prevalent compared to AFB_1_ and AFB_2_ (Figure 4C,D). Total AFs concentrations reached their highest levels under combined elevated temperature and variable humidity conditions (mainly below 70%), demonstrating a combined influence of these climatic factors on AFs production (Figure 4E).

The broader relationship between climatic factors and various mycotoxins further highlights the critical role of temperature and humidity in the maize growing season on fungal development and toxin biosynthesis. The concentrations of total FB, including FB_1_ and FB_2_, were highest under moderate humidity (65–70%) and temperatures between 18 °C and 22 °C, with FB_1_ showing higher concentrations than FB2 (Figure 5A–C). DON concentrations peaked under moderate humidity (~65%) and cooler temperatures (16–18 °C), as illustrated in Figure 5D. ZEN concentrations increased sharply under high humidity conditions (>70%) and temperatures between 18 °C and 20 °C (Figure 5E). T-2 toxin and HT-2 toxin contamination levels were generally lower, with peaks occurring at cooler temperatures (14–16 °C) and moderate humidity (~65%) (Figure 5F,G). Cumulative *Fusarium* mycotoxin concentrations, including DON, ZEN, FB_1_, and FB_2_, showed significant peaks under moderate humidity and temperatures between 18 °C and 20 °C (during the maize growth phase, Figure 5H). Total mycotoxin levels were highest under conditions of elevated humidity (>70%) and moderate temperatures (18–22 °C), indicating the compounded impact of climatic factors of the growing season on mycotoxin contamination (Figure 5I).

Mycotoxin co-contamination analysis, which examines the number of mycotoxins per sample, emphasises the interplay between climatic conditions during crop growth and fungal proliferation. The highest levels of co-contamination, with up to 10 mycotoxins per sample, were observed under high relative humidity (70–75%) and elevated temperatures (20–22 °C), underscoring the synergistic role of these climatic factors in promoting multi-mycotoxin contamination (Figure 6). Intermediate co-contamination levels, involving 5–7 mycotoxins per sample, occurred under moderate humidity (65–70%) and temperatures between 18 °C and 20 °C. In contrast, minimal co-contamination was recorded under lower humidity conditions (<65%) and cooler temperatures (<18 °C), suggesting less favourable conditions for fungal proliferation and diversity of toxin production (Figure 6). These findings collectively demonstrate the significant influence of specific temperature and humidity conditions on the prevalence of AFs, *Fusarium* mycotoxins, and co-contamination, highlighting the substantial risks of warm and humid growing seasons in maize production.

### 2.4. Implications for Food and Feed Safety

Table 4 shows the maximum and guidance levels (ML/GL) of regulated mycotoxins in maize intended for human and animal consumption, according to the European and Serbian legislations [30,31,32,33,34,35,36,37]. The table also indicates the non-compliance rates for regulated mycotoxins in maize samples harvested in Serbia in 2021–2023 (according to current Serbian and European legislation). AFs, OTA, FBs (FB_1_ + FB_2_), and ZEN presented non-compliance levels for human consumption. Concerning the compliance levels for animal consumption, some of the analysed samples presented concentrations of AFB_1_ above the maximum limit and concentrations of OTA and the sum of the toxins T-2 and HT-2, which were above the guidance level. In 2021, FBs were the mycotoxins that had a high occurrence above the compliance values for human consumption. The samples from 2022 present higher non-compliance rates for AFs and OTA. During 2022 and 2023, <1.5% of the samples exceeded the maximum EU limit for food. The high noncompliance levels of AFs and FBs in analysing Serbian maize raised serious food and feed safety concerns. According to European Union regulations, AB_1_ contamination in food products should not exceed five µg/kg for human consumption. However, in 2022, 55.4% of maize samples exceeded this limit (Table 4), with some reaching levels as high as 527 µg/kg (Table 2). This poses a substantial risk to public health, especially considering that AFs are potent carcinogens with a well-established link to liver cancer. Regarding feed safety, 40.5% of the samples exceeded the regulatory limit of 20 µg/kg set for animal feed, which could result in bioaccumulation in livestock and subsequent exposure to humans through meat and dairy products. For the toxins T-2 and HT-2, the samples above the ML for human consumption are 1.4%, 0.6%, and 0.6% in 2021, 2022, and 2023, respectively, and 0.6% of the samples of 2022 presented values over the GL for feed.

## 3. Discussion

The occurrence of regulated mycotoxins in Serbian maize samples during 2021–2023 revealed significant temporal and geographical variability, driven mainly by climatic conditions. AFB_1_ exhibited the highest variability, with a dramatic increase in contamination in 2022 (73.2% occurrence) compared to 2021 (18.5%) and 2023 (34.1%). These fluctuations are likely linked to environmental factors, as 2022 was characterised by higher humidity and rainfall during the growing season, creating favourable conditions for *Aspergillus* species, which produce AFs. The peak contamination exceeding 500 µg/kg poses a significant health risk, considering the carcinogenic nature of AFs and the strict regulatory MLs for both human and animal consumption [30,32,33]. AFs, especially AFB_1_, are among the most potent carcinogens known, with a well-documented association with hepatocellular carcinoma (HCC) in humans [38,39]. Chronic exposure to AFB_1_ can also impair immune function and exacerbate malnutrition, particularly in children in regions with high dietary maize consumption [40,41,42,43]. For animals, AFs can cause reduced growth performance, liver damage, and immune suppression, with dairy cattle at risk of producing AFM1-contaminated milk [44,45,46,47]. The high non-compliance rates in 2022 highlight the need for stringent monitoring during wet growing seasons to prevent public health concerns, such as hepatocellular carcinoma in humans and losses in animal agriculture. While possible, it is not likely for farm animals to develop carcinoma, primarily due to their limited lifespans, which often do not allow sufficient time for cancer development [48,49]. Besides human health, AFs can also adversely affect animal health. As a proven case report from Pakistan, in 2023, there was a 75% abortion rate and a significant loss of milk yield (−18.8%) at a farm in Pakistan after the inclusion of concentrate feed. Tests of the feed showed that AFB_1_ contamination levels of 165 µg/kg DM were 29 times higher than the limit of 5.68 µg/kg DM as regulated by the European Union [50]. The high levels of mycotoxin contamination observed, particularly in 2022, seriously affect food and feed safety in Serbia. AFs, for instance, exceeded the EU and Serbian regulatory limit of 5 µg/kg for human consumption in over 55% of the samples analysed in 2022. This group of mycotoxins represents a significant public health risk, particularly considering their carcinogenic properties and their role in liver cancer development. The presence of AFB_2_, AFG_1_, and AFG_2_ further complicates the risk profile, as these toxins could exhibit additive or synergistic toxic effects when combined with AFB_1_. The high levels of total AFs observed in 2022 represent a significant public health concern, particularly for vulnerable populations in Serbia.

FBs, particularly FB_1_ and FB_2_, were detected consistently across all three years, with a notable reduction in contamination levels in 2023. The high levels of FBs in 2021 may be attributed to optimal growing conditions for *Fusarium* species, particularly during conditions with moderate temperature and moisture levels. FBs (especially FB_1_) were the mycotoxins with the highest levels detected. Contamination levels of FBs tend to drive the total concentration of *Fusarium* mycotoxins and total mycotoxins (Supplementary Figure S1; Supplementary Table S3). Despite the lower occurrence in 2023, contamination with FBs remained a concern, particularly for feed safety, where concentrations often exceeded the 4000 µg/kg limit for human consumption. FBs, produced by *Fusarium* species, are linked to oesophageal cancer and neural tube defects in humans. Their interference with sphingolipid metabolism underscores their systemic toxicity [51,52,53]. In animals, FBs cause leukoencephalomalacia in horses [54,55], pulmonary oedema in swine [56], and impaired liver function in poultry [57,58]. The significant non-compliance in 2021 and 2022 highlights the need for targeted mitigation strategies during years with moderate humidity. It is also interesting to note that they were revealed to be the cause of Equine leukoencephalomalacia or “Moldy corn poisoning”, which causes severe neurological symptoms and, sometimes, sudden death [59]. One case described an outbreak at a farm in Brazil in 1996 where four horses were fed contaminated food rations and died 18–30 hours after showing clinical signs. Samples of corn were collected from the site, and the amount of FB_1_ found was at least five times higher than the known toxic concentration for horses [60].

The *Fusarium*-produced toxins, DON and ZEN, were less frequently detected but still present at concerning levels, particularly in 2021 and 2022. Although lower in 2023, DON contamination remains a significant problem due to its immunotoxic effects. ZEN, an estrogenic mycotoxin, peaked in 2022 with average contamination levels reaching 357.6 µg/kg, further highlighting the potential health risks associated with *Fusarium* toxins in Serbian maize. DON, also known as vomitoxin, induces acute gastrointestinal distress and immune suppression in humans, with possible long-term impacts on growth and development [61,62,63,64]. In animals, DON reduces feed intake and causes vomiting, particularly in swine [65]. This study’s absence of DON non-compliance suggests favourable conditions for its management in Serbian maize, but vigilance remains essential due to its variability across regions. Zearalenone is another important mycotoxin; it is characterised by having similarities to natural oestrogen and, as such, can lead to numerous hormonal and reproductive diseases, especially in pigs. It is mainly found in temperate regions, and commonly contaminates maize [63]. In animals, it can cause fertility issues such as infertility, reduced incidence of pregnancy, and even abortions [66,67].

Ochratoxin A (OTA) and trichothecenes (HT-2 and T-2) were detected at lower frequencies, yet their sporadic presence, particularly in 2022, cannot be overlooked. OTA contamination in 29.8% of the samples in 2022 suggests that the toxin, though less common, still poses a risk, particularly under specific environmental conditions. OTA is nephrotoxic and has been classified as a possible human carcinogen by the International Agency for Research on Cancer (IARC). In humans, it is linked to Balkan endemic nephropathy and urinary tract cancers [68,69]. OTA causes reduced feed efficiency, renal damage, and immunosuppression in animals, particularly in pigs and poultry [70]. The overall presence of HT-2 and T-2 toxins was minimal, but the data suggest that environmental shifts could still provoke sporadic outbreaks [71]. The sporadic non-compliance observed suggests that targeted interventions during wetter years could mitigate these risks. These trichothecenes are among the most acutely toxic mycotoxins, causing gastrointestinal lesions, immunosuppression, and neurotoxicity in humans [71]. In animals, they lead to feed refusal, reduced weight gain, and haemorrhagic syndromes [72,73]. Although detected at low levels, their severe toxicity warrants strict surveillance.

The co-contamination of maize samples with multiple mycotoxins was a notable finding in this study, particularly in 2022. Over 72% of the maize samples were contaminated with at least two mycotoxins, predominantly AFs and FBs (AFB_1_ and FB_1_). This co-occurrence is of great concern due to potential additive or synergistic toxic effects, which can exacerbate the impact on both human and animal health [74,75,76,77,78]. It has been proven that low doses of mycotoxin mixtures below EU regulatory limits can negatively affect the productive performance of animals [79]. Studies have shown that when AFs and FBs are present together, they can intensify the risks of hepatotoxicity and immunosuppression [80]. The high prevalence of these two mycotoxins in combination underscores the importance of monitoring co-contamination and assessing the cumulative risks of consuming maize contaminated with multiple toxins. This high incidence of co-contamination is concerning due to the potential synergistic effects of these toxins on both human and animal health [74,75]. In addition to AF-FB combinations, detecting DON and ZEN alongside FBs in a small percentage of samples further complicates the safety profile of maize, as these toxins are linked to gastrointestinal and reproductive issues. The frequent co-contamination of maize with different groups of mycotoxins highlights the need for multi-mycotoxin detection methods and integrated risk assessments that consider the combined exposure to several mycotoxins simultaneously, as suggested in recent studies [5,12,81,82,83].

Climatic factors were crucial in shaping the mycotoxin contamination pattern across the three-year study period. The strong correlation between rainfall and AF contamination highlights the vulnerability of Serbian maize to climatic variations. The year 2022, which experienced higher-than-average rainfall during the growing season, also had the highest levels of AFs. This is consistent with the known influence of moisture on *Aspergillus* growth and AFs production [29,84]. Similarly, the correlation between temperature and FBs levels suggests that higher temperatures, particularly during the growing season, promote the growth of *Fusarium* species, which thrive in warmer environments. The climatic implications of this study are particularly important in the context of global climate change [85,86,87]. As temperatures rise and rainfall patterns become more erratic, the risk of mycotoxin contamination in maize is expected to increase [84]. This trend underscores the need for ongoing monitoring and predictive modelling to anticipate high-risk years based on weather conditions. Strategies to mitigate mycotoxin contamination, such as altering planting and harvesting times, using resistant maize varieties, and implementing better post-harvest handling and storage, must be prioritised in regions vulnerable to climate variability. Thus, the findings from this study underscore the critical role of climatic factors in shaping mycotoxin contamination patterns in Serbian maize [5,8,26,29]. The high incidence of co-contamination, coupled with the elevated levels of AFs and FBs, particularly in 2022, calls for urgent action to improve mitigation strategies, ensure compliance with safety regulations, and protect both public health and agricultural exports [8,29,84].

The results demonstrate a clear relationship between climatic factors, particularly temperature and relative humidity, and AF contamination in maize. These findings align with previous research that highlights the susceptibility of maize to AF contamination under warm and humid conditions [26]. The peak contamination of AFB_1_ at a monthly average temperature of 20–22 °C and humidity levels > 70% reflects the ecological requirements of *Aspergillus flavus*, the primary producer of AFs. Previous studies have confirmed that *A. flavus* thrives in warm, moist environments, with optimal AF production occurring at temperatures of 25–30 °C and high relative humidity [9,88]. The significant contamination levels observed in 2022 can be attributed to the wetter growing season that year, which created ideal conditions for fungal growth and AF production. The patterns observed for AFB_2_, AFG_1_, and AFG_2_ suggest that these toxins are secondary metabolites with production highly dependent on specific ecological conditions [89,90]. AFB_2_, in particular, mirrors AFB_1_ trends but at lower levels, indicating a co-dependent production process by *A. flavus* and *A. parasiticus*. The cumulative increase in AF concentrations under air dryness (low humidity) and temperature highlights the compounded risk of multi-toxin contamination during unfavourable climatic conditions [91,92,93].

The findings underscore the critical role of temperature and humidity in influencing mycotoxin contamination in maize. The dependency of fungal growth and mycotoxin production on specific climatic conditions is well-documented in the literature, with *Fusarium* and *Aspergillus* spp., demonstrating distinct ecological preferences [94]. The correlation between AF concentrations and climatic conditions underscores the vulnerability of maize production to climate variability. As global temperatures rise and rainfall patterns become more erratic, the risk of AF contamination is expected to increase [95]. The findings emphasise the need for predictive models integrating climatic data to forecast high-risk periods for AF contamination. Such tools could enable farmers to implement timely interventions, such as adjusting planting and harvesting schedules or adopting drought-resistant maize varieties [88]; however, it is essential to mention that the monthly averages are not the ideal values to develop valid models, because the variations during the days can influence the mycotoxin synthesis. FBs are primarily produced by *Fusarium verticillioides* and *F. proliferatum*, which thrive under moderate temperatures (18–22 °C) and humidity levels (60–70%). These conditions were prevalent in Serbian maize fields during the study period, particularly in 2021 and 2022, leading to elevated FB concentrations. Previous research has shown that FB contamination is strongly correlates with rainfall during the growing season, which creates a favourable environment for *Fusarium* infection [96,97,98]. The optimal temperature for *F. graminearum* and *F. culmorum* generated DON production is between 25 and 30 °C, depending on the incubation length [99]; however, to maintain toxin production, the temperature should not drop below 11 °C. DON production generally requires a much narrower raw and temperature range as compared to the *Fusarium* growth. The available data on ecological profiles of ZEN production are scarcer [100]. Most of the *F. verticillioides* isolates primarily produce FB_1_ and smaller amounts of FB_2_, FB_3_, and FB_4_. The temperature range facilitating the growth of the above mould is far wider than the range needed to produce FBs. The mould in question grows at temperatures of 4–37 °C, the optimum thereby approximating 30 °C, while FBs are mainly produced within the temperature range of 10–37 °C, the optimum thereby being 15–30 °C [101,102]. DON, produced by *Fusarium* spp. (e.g., *F*. *graminearum*), is associated with calm and wet conditions during the flowering and early grain-filling stages. The observed peak DON concentrations at 16–18 °C temperatures align with studies indicating that *Fusarium* thrives under such climatic conditions [103,104]. Although DON levels remained below regulatory thresholds in most samples, its immunosuppressive effects make it a critical mycotoxin to monitor [28]. The sharp increase in ZEN levels under high humidity (>70%) is consistent with the environmental preferences of *Fusarium* species. ZEN is an estrogenic mycotoxin with significant implications for reproductive health in livestock, particularly swine. The findings highlight the importance of humidity levels during the maize growing season on ZEN contamination [103]. T-2 and HT-2 toxins, trichothecenes produced by various *Fusarium* species, were less prevalent in the samples but showed distinct peaks under cooler temperatures (14–16 °C). These toxins are known for their acute toxicity and pose significant risks to poultry and livestock [105]. The high concentrations of total mycotoxins observed under elevated humidity and moderate temperatures emphasise the compounded risks posed by multiple mycotoxins. Co-occurrence of FBs, DON, and ZEN, as observed in this study, has been shown to exacerbate toxicological effects in both humans and animals [106]. The synergistic interactions between these mycotoxins can amplify their hepatotoxic, immunosuppressive, and reproductive health impacts [20,107]. The findings in Figure 5 reinforce the critical role of climatic factors in influencing mycotoxin co-contamination levels in maize. Warm temperatures and high humidity create an ideal environment for the proliferation of multiple mycotoxigenic fungi, such as *Aspergillus*, *Fusarium,* and *Penicillium* species, which can produce a wide range of mycotoxins simultaneously [94]. As global temperatures rise and rainfall patterns become increasingly erratic, the risk of mycotoxin contamination in maize is expected to grow. Climate change models predict an expansion of regions favourable for *Fusarium* and *Aspergillus* proliferation, leading to higher mycotoxin burdens [95].

The observed contamination levels pose significant challenges for food and feed safety, particularly in regions where maize is a dietary staple. Mycotoxin contamination exceeding regulatory limits can lead to severe economic losses due to trade restrictions and health impacts. Ensuring compliance with international safety standards requires robust monitoring systems and integrated mitigation strategies to address the dual threats of individual and cumulative mycotoxin exposure. These findings could have significant economic consequences for Serbia, a major maize producer and exporter. Mycotoxin contamination above allowable limits can lead to trade restrictions, reducing the competitiveness of Serbian maize in international markets. This underscores the need for stricter regulatory control, improved storage practices, and better post-harvest management to ensure compliance with food and feed safety standards. The ability to meet these standards is vital for protecting public health and maintaining Serbia’s position in the global maize market. It is important to note that while regulations exist for individual mycotoxins, such as AF, FBs, DON, and ZEN, there are currently no specific legal limits addressing the co-occurrence of two or more mycotoxins in food or feed [74]. This regulatory gap poses a significant challenge for food safety, as co-contamination is frequently observed, particularly in maize, as evidenced in this study of Serbian maize from 2021 to 2023.

The implications for export markets are equally concerning. Mycotoxin contamination exceeding international safety standards can lead to trade restrictions, adversely affecting Serbia’s maize export market. FB contamination also posed a significant threat, with 22.5% of samples in 2022 surpassing the maximum limit of 4000 µg/kg for human consumption, further complicating compliance with EU and global trade standards. These findings highlight the need for enhanced mycotoxin monitoring and mitigation strategies to maintain the competitiveness of Serbian maize in international markets and ensure food and feed safety domestically [108]. The study focused on regulated mycotoxins (AFs, Fs, DON, ZEN, HT-2, T-2, OTA). However, other frequent non-regulated mycotoxins or emerging mycotoxins, which may also pose health risks, were not analysed. Including a broader range of mycotoxins offers a more complete risk assessment [109]. Thus, another broad number of toxins is required for a complete analysis. Considering the diversity of co-occurring (emerging) toxins and metabolites derived from species of *Alternaria*, *Fusarium*, *Penicillium*, *Aspergillus*, and *Claviceps* ergot alkaloids, the levels of total mycotoxin and toxicological profile presented in this study are just the “tip of the iceberg” [110]. The importance of addressing the simultaneous presence (also referred to as real-world mixtures) of natural and synthetic chemicals is a key focus for mixture toxicologists [111,112,113]. With over 500 identified fungal toxins and their associated metabolites, along with the well-established co-occurrence of various mycotoxins, multi-metabolite analyses have emerged as practical tools to provide a more precise understanding of these contaminant mixtures within the food and feed supply chain [74,114,115,116].

While our results primarily reflect lowland regions, which constitute the most significant portion of maize production in the country, they underline the importance of considering topographical factors when assessing contamination risks. Vojvodina, a flat and low-lying agricultural area, accounted for the majority of the sampled fields, while fewer samples were collected from hilly and mountainous regions. Consequently, the data presented here are more representative of lowland maize cultivation, which may limit the generalisability of the findings to higher altitudes. This imbalance in sampling distribution highlights the need for future studies to adopt a more balanced approach, including increased sampling at higher elevations. Such efforts would help clarify potential variations in contamination risks associated with diverse topographies. Addressing these gaps will contribute to a more holistic understanding of the impact of geographical factors on maize contamination and support the development of tailored mitigation strategies for different production environments. The climatic data for this study were obtained from the nearest weather stations to each sampling location. To enhance the representativeness and precision of future research, we advocate for using new technologies that allow the collection of field-specific climatic data. This would significantly improve both the quality of the dataset and the reliability of statistical analyses and possible predictive models. However, the high costs associated with collecting such detailed climatic data, such as specialised equipment, remain a limiting factor. Moreover, the study did not account for variations in agricultural practices, such as pesticide use, crop rotation, or irrigation methods, which could influence fungal growth and mycotoxin contamination. Including these factors could provide insights into the role of farm management in reducing mycotoxin risks. While the study focused on mycotoxin contamination during the growing season, post-harvest storage conditions were not analysed. Mycotoxin levels can increase during storage if proper conditions (e.g., moisture control) are not maintained [117,118]. Future studies should include assessing storage practices to understand the contamination cycle better. By addressing these limitations, future research can build on the findings of this study to provide a more comprehensive assessment of mycotoxin contamination risks and develop more effective mitigation strategies [118].

The results of this study point to several key areas for future research and action. First, the ongoing impact of climate change on mycotoxin contamination should be a major focus, with a particular emphasis on developing predictive models that integrate geo-climatic data to forecast high-risk periods for mycotoxin contamination [22,28,29]. These models could serve as valuable tools for farmers and policymakers to implement preventive strategies during years with adverse climatic conditions. Second, the high incidence of co-contamination necessitates a more integrated approach to mycotoxin risk assessment. Future studies should explore the toxicological interactions between co-occurring mycotoxins and their combined effects on health [74]. This will provide a more comprehensive understanding of the risks posed by multi-mycotoxin contamination and help refine regulatory frameworks to account for cumulative exposure. Additionally, there is a need for the development of more resilient maize varieties that are less susceptible to fungal infection and mycotoxin contamination. Moreover, the use of strategies for reducing the risk and adverse effects in animal feeds, such as anti-mycotoxin feed additives (like aluminosilicates, e.g., bentonite and clinoptilolite), can also contribute to the mitigation of mycotoxin adverse impacts on the profitability and safety of the feed and food chains [119,120,121,122,123].

Biological decontamination strategies utilise microorganisms such as algae, moulds, yeasts, and bacteria. These microorganisms can bind to, modify, or break down mycotoxins into less toxic compounds in certain foods and feed through processes like decarboxylation, acetylation, hydrolysis, glycosylation, or deamination [124]. For example, some bacteria, plants, moulds, and yeasts are capable of converting ochratoxin A into phenylalanine [125]. Enzymes and microorganisms can also be incorporated into feed to facilitate mycotoxin degradation or detoxification in the gastrointestinal tracts of ruminants [126]. Among the most commonly used microorganisms for decontaminating mycotoxins are yeasts and lactic acid bacteria, which can reduce toxin levels by binding to their cell surfaces or transforming them into less harmful substances. Furthermore, enzymatic catalysis shows excellent potential for effectively decontaminating mycotoxins [127].

## 4. Conclusions

This study demonstrated the significant impact of climatic factors on the occurrence and levels of regulated mycotoxins in Serbian unprocessed maize grains harvested from 2021 to 2023. Aflatoxins, particularly AFB_1_, posed the most severe food and feed safety risks, with contamination levels exceeding the European regulatory limits by up to 105.4 times. Fumonisins also showed high contamination frequencies, particularly in 2021, with levels decreasing in subsequent years. Co-contamination with multiple mycotoxins was common, raising concerns about potential synergistic toxic effects. The strong correlations between climatic conditions and mycotoxin levels underline the necessity of continuous monitoring and improved mitigation strategies to ensure food and feed safety. Developing predictive models to forecast high-risk periods and implementing adaptive agricultural practices are essential to mitigate mycotoxin contamination risks and safeguard public and animal health.

## 5. Materials and Methods 

### 5.1. Sample Collection

**Figure 7:**
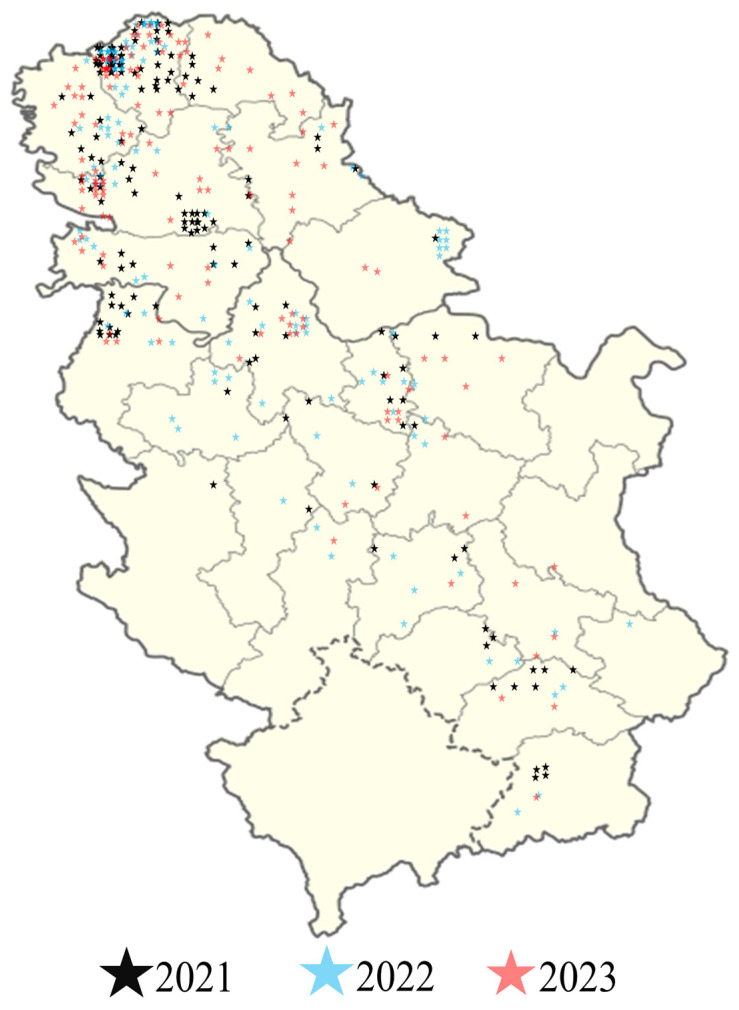
Map of the Republic of Serbia illustrates the locations of the analysed maize samples.

Samples of unprocessed maize grains from various agricultural regions across Serbia were collected during the harvest seasons of 2021, 2022, and 2023. The overall number of samples analysed in this study was 548: 216 (39.4%), 168 (30.7%), and 164 (29.9%) collected in 2021, 2022, and 2023, respectively. The maize samples were collected in fields located in the 25 localities/districts (Banatski Karlovac, Belgrade, Ćuprija, Kikinda, Kopaonik, Kragujevac, Kraljevo, Kruševac, Leskovac, Loznica, Negotin, Niš, Novi Sad, Priština, Palić, Požega, Priština, Sjenica, Smederevska Palanka, Sombor, Sremska Mitrovica, Valjevo, Veliko Gradište, Vranje, and Zrenjanin). The sampling covered different climatic regions, with a comprehensive representation of the maize production from the Region of Vojvodina, City of Belgrade, and Southern and Eastern Serbia (Figure 7). Maize fields from which samples were collected were situated at varying altitudes, reflecting the diverse topography of Serbia. However, Vojvodina, located in Northern Serbia and at lower altitudes, presented a higher representation of samples from 408 fields (74.45%). In contrast, Šumadija and Western Serbia, known for their more hilly and elevated terrain, accounted for 59 fields (10.77%). Southern and Eastern Serbia, encompassing both low-land and mountainous areas, contributed 57 fields (10.40%). The City of Belgrade, with a mix of urban and peri-urban agricultural zones at relatively low altitudes, comprised 24 fields (4.38%). The samples of unprocessed maize grains were collected by farmers immediately after the harvest, before post-harvest cleaning or drying processes, directly from the harvester. Each composite sample consisted of at least 50 subsamples and weighed at least 10 kg. The sample was manually mixed, and approximately 1 kg was sent to the Patent Co. analytical laboratories for analysis. To maintain the representativeness of the collected samples, once they were in the quality control laboratory of PATENT CO. in Mišićevo, Serbia, the samples were homogenised, quartered (using a Riffle sample divider), and milled by trained laboratory technicians and stored at −20 °C until the analysis.

### 5.2. Multi-Mycotoxin Analysis

#### 5.2.1. Sample Preparation

Upon collection, maize samples were immediately transported to the laboratory for preparation and analysis. The samples were first finely ground and homogenised using a laboratory ultra-centrifugal mill (model ZM200, producer Retsch, Haan, Germany) to ensure a uniform particle size (<0.5 mm). A 5.0 ± 0.001 g sub-sample of each maize sample was weighed and placed in a conical tube. Approximately 20 mL of extraction mixture I (80% Acetonitrile, 19.9% Water, and 0.1% Formic acid) and the lid were closed. The tube was thoroughly mixed on an orbital shaker at 200 rpm for 1 h at room temperature. The tube was then centrifuged at 4200× *g* for 5 min, and the supernatant was removed to another 50 mL conical tube. A 20 mL aliquot of the extraction mixture II (20% Acetonitrile, 79.9% Water, and 0.1% Formic acid) was added to the residue, and the mixture was again extracted by placing it on an orbital shaker at 200 rpm for 30 min at room temperature. This mixture was centrifuged at 4200× *g* for 5 min. The two supernatants of the two extraction steps were combined and again centrifuged at 4200× *g* for 5 min. A 500 µL aliquot was prepared by filtering the extract through a membrane syringe filter into a glass vial. To compensate for the matrix effects during electrospray ionisation, the extracts were mixed with (13C)-labelled internal standards for each group of mycotoxins (13C AFB_1_, 13C DON, 13C ZEN, 13C OTA, 13C FB_1_, and 13C T-2) (Romer Labs^®^, Tulln, Austria).

#### 5.2.2. LC-MS/MS Parameters

The quantification was carried out using a highly sensitive and specific method for multi-mycotoxin detection using liquid chromatography-tandem mass spectrometry (LC-MS/MS) triple quad (Agilent 6460 series, Agilent Technologies, Santa Clara, CA, USA). The analysis targeted several key mycotoxins, including AFB_1_, AFB_2_, AFG_1_, AFG_2_, OTA, ZEA, DON, FB_1_, FB_2_, T-2, and HT-2 toxins that are all regulated in the EU in feed by EU Directives 2002/32/EC, 2006/576/EC, and 2013/165/EU. The method was successfully validated on maize, compound feed, wheat, barley, soybean meal, wheat bran, sunflower meal, and total mixed rations (TMR). The mass spectrometer was operated in multiple reaction monitoring (MRM) mode with both positive and negative ionisation, depending on the mycotoxin. Ionisation was achieved using an electrospray ionisation (ESI) source. The following parameters were used: capillary voltage of 3500 V, sheath gas temperature of 350 °C, and sheath gas flow of 11l/min. Specific MRM transitions for each mycotoxin were monitored based on their characteristic precursor and product ions. Quantification was performed using external calibration curves prepared from standard solutions of each mycotoxin, with correlation coefficients greater than 0.99. The LOQ for each mycotoxin is shown in Table 5, with values ranging from 0.4 µg/kg for AFs to 64 µg/kg for DON. Recoveries for all analytes ranged from 70% to 120%, and the relative standard deviation (RSD) for repeatability was below 15% (2.5% and 13.4%). The method’s performance parameters were obtained by in-house validation. The Relative Standard Deviation (RSDr) of the method was between 2.5% and 13.4%, and the recoveries were between 62% and 115% for all analytes. The LOQs for analysed mycotoxins are in Table 5 [13,128]. The mycotoxin identification was performed according to the document SANTE/12089/2016 [129]. The analytic method’s performance was validated according to SANTE/12682/2019 [130]. Key parameters assessed included linearity (0.95 ≤ R2), limit of quantification (LOQ), recovery, and repeatability [13,130].

### 5.3. Climate Data

The climatic data of the crop locations, including altitude, average air temperature of the month of sampling, average air temperature, relative humidity, and the sum of precipitation (accumulated rainfall). The climatic data were collected from the nearest climatological station of the Republic Hydrometeorological Service of Serbia for the entire period 2021–2023. Sample-specific climatic data were merged with the mycotoxin contamination of the respective year as an integrated dataset for statistical analysis. The maize growing season was considered the average value from June to September in the 3 different years. The average relative humidity, rainfall, and air temperature of the maize growing season and harvest month were matched with the mycotoxin concentrations of each sample based on the municipalities/districts of the farms (provided by the Republic Hydrometeorological Service of Serbia) (https://www.hidmet.gov.rs/eng/meteorologija/moss_naslovna.php). Climatic data, including temperature, humidity, and rainfall (growing season and harvest month), were checked and recorded for the correlation and regression analyses.

### 5.4. Statistical Analysis

Descriptive statistics, including occurrences and concentration values (mean, median, minimum, and maximum), were calculated using only the data above the limit of quantification (LOQ). Values below the LOQ were considered non-detectable. All concentrations are expressed in µg/kg (parts per billion—ppb), adjusted to a moisture content of 12%. In this study, the occurrence of the total (sum) mycotoxins was calculated by determining the number of samples in which at least one mycotoxin from a specific group (e.g., total aflatoxins, total fumonisins, total trichothecenes) was detected above the limit of quantification (LOQ). The total concentration of a mycotoxin group was obtained by summing the concentrations of all individual mycotoxins detected within that group (e.g., AFB_1_ + AFB_2_ + AFG_1_ + AFG_2_ for total aflatoxins). Using GraphPad Prism (version 10.1, GraphPad Software, San Diego, CA, USA), the Kruskal–Wallis test was employed to determine significant differences among the annual contamination levels. For co-occurrence analysis, a matrix was constructed in Microsoft Excel to capture the detection frequencies of common mycotoxin combinations in maize, and a heatmap was generated using GraphPad Prism. Spearman’s correlation analysis assessed the relationship between mycotoxin concentrations and geo-climatic factors (temperature, humidity, rainfall, and altitude). Three-dimensional mesh graphs representing the relationship among contamination levels of mycotoxins and the total number of mycotoxins per sample with relative humidity (%) and temperature (°C) during the growing season (June to September) were plotted using SigmaStat (Version 4.0; Systat Software, San Jose, CA, USA). Correlations with a significance level of *p* < 0.01 were further analysed to evaluate the influence of climatic conditions on contamination levels. Correlation strengths were interpreted following Schober et al. (2018) [131], where coefficients were categorized as “very strong” (0.90–1.00), “strong” (0.70–0.89), and “moderate” (0.40–0.69).

## Figures and Tables

**Table 1:** Description of the climatic factors and altitude of the localities of the analysed maize samples harvested in Serbia during 2021–2023.

Parameter	Year	Average ± SD			Range		
Altitude (m.a.s.l.)		266.3	±	423.2	42	–	2017
Temperature (harvest’s month ^1^) (°C)	2021	16.9	±	2.1	9.2	–	19.4
	2022	16.2	±	2.0	8.5	–	18.1
	2023	19.8	±	2.2	11.9	–	22.2
Temperature (growing season ^2^) (°C)	2021	21.2	±	2.2	13.0	–	23.6
	2022	21.2	±	2.3	12.6	–	23.4
	2023	21.5	±	2.2	13.4	–	23.7
Temperature (annual) (°C)	2021	11.7	±	1.9	4.3	–	13.8
	2022	12.4	±	2.0	5.0	–	14.5
	2023	12.9	±	2.0	5.4	–	14.8
Humidity (harvest’s month ^1^) (%)	2021	76.3	±	4.4	70.2	–	86.5
	2022	75.0	±	4.4	63.0	–	80.1
	2023	70.8	±	4.9	62.0	–	85.4
Humidity (growing season ^2^) (%)	2021	62.2	±	4.8	54.1	–	73.0
	2022	64.4	±	5.4	54.5	–	73.9
	2023	70.5	±	6.0	55.5	–	79.5
Rainfall (harvest month ^1^) (mm)	2021	70.2	±	22.3	38.6	–	107.5
	2022	11.6	±	8.6	1.5	–	31.0
	2023	13.5	±	7.5	1.1	–	30.6
Rainfall (growing season ^2^) (mm)	2021	194.9	±	56.2	89.8	–	306.6
	2022	305.5	±	87.3	189.7	–	530.1
	2023	298.7	±	73.4	158.8	–	448.8

^1^ September average; ^2^ Average of the period (June–September). The Republic Hydrometeorological Service of Serbia provided the climatic data. m.a.s.l.: meters above sea level.

**Table 2:** Occurrence and contamination levels of regulated mycotoxins detected in maize samples collected in Serbia in 2021–2023.

Mycotoxin	Year	Occurrence (%) ^1^	Concentration (µg/kg) ^2^						Kruskal–Wallis Test *p*-Value
			Average (±SD)			Range			
	2021	18.5	10.0 ^B^	±	16.5	0.4	–	101	<0.0001 *
Aflatoxin B_1_	2022	73.2	80.6 ^A^	±	115.3	0.5	–	527	
	2023	34.1	29.2 ^AB^	±	43.3	0.6	–	218	
	2021	9.3	2.5 ^B^	±	4.0	0.4	–	17	<0.0001 *
Aflatoxin B_2_	2022	54.8	26.3 ^A^	±	57.7	0.5	–	390	
	2023	23.2	6.5 ^B^	±	11.7	0.4	–	61.6	
	2021	1.4	5.3	±	2.9	1.4	–	8.4	0.6260
Aflatoxin G_1_	2022	23.8	6.9	±	9.7	0.5	–	51.8	
	2023	0	nd						
	2021	0	nd						–
Aflatoxin G_2_	2022	4.7	37.3	±	73.7	0.6	–	231	
	2023	0	nd						
Total of aflatoxins	2021	18.5	12.0 ^B^	±	20.0	0.4	–	118	<0.0001 *
	2022	73.2	105 ^A^	±	162.2	0.5	–	897	
	2023	34.1	33.6 ^B^	±	52.1	0.6	–	245	
	2021	2.3	37.6	±	34.6	4.8	–	102	0.8173
Ochratoxin A	2022	29.8	65.2	±	89.5	1.6	–	325	
	2023	3.7	76.6	±	66.1	2.6	–	190	
	2021	3.2	40.0	±	13.9	20.7	–	65.5	0.3596
Zearalenone	2022	2.4	357.6	±	347	21.8	–	851	
	2023	1.2	221.6	±	164	57.2	–	386	
	2021	87.5	291	±	188	95.3	–	606	0.6973
Deoxynivalenol	2022	87.5	304	±	248	64.4	–	904	
	2023	70.7	222	±	123	71.0	–	474	
	2021	87.1	4532 ^A^	±	4617	45.0	–	28,781	<0.0001 *
Fumonisin B_1_	2022	87.6	2202 ^B^	±	2158	42.3	–	11,775	
	2023	70.7	885 ^C^	±	981	43.4	–	5731	
	2021	81.9	1653 ^A^	±	2115	45.1	–	15,479	<0.0001 *
Fumonisin B_2_	2022	79.2	637 ^B^	±	689	40.4	–	3868	
	2023	59.8	280 ^C^	±	291	42.2	–	1848	
Total of fumonisins	2021	87.5	6080 ^A^	±	6659	45.0	–	44,260	<0.0001 *
	2022	87.5	2778 ^B^	±	2816	42.3	–	15,643	
	2023	72.0	1102 ^C^	±	1249	43.4	–	7168	
	2021	5.1	48.7	±	28.5	18.9	–	114	0.0690
HT-2	2022	6.5	54.7	±	98.2	9.8	–	362	
	2023	1.2	84.3	±	31.7	52.6	–	116	
	2021	2.8	49.0	±	23.8	15.1	–	86.4	0.4655
T-2	2022	4.1	36.4	±	31.1	9.3	–	107	
	2023	1.2	27.9	±	10.3	17.6	–	38.1	
Total of HT-2 and T-2	2021	5.1	75.4	±	55.2	18.9	–	200	0.1801
	2022	7.1	71.4	±	123	9.8	–	469	
	2023	1.8	74.8	±	41.7	17.6	–	116	
Total of Trichothecenes ^3^	2021	10.1	196	±	179	18.9	–	606	0.6772
	2022	14.8	241	±	247	9.8	–	904	
	2023	13.4	202	±	126	17.6	–	474	
Total of *Fusarium* toxins ^4^	2021	89.4	5978 ^A^	±	6639	20.7	–	44,289	<0.0001 *
	2022	88.7	2791 ^B^	±	2799	42.3	–	15,643	
	2023	72.0	1144 ^C^	±	1262	43.4	–	7214	
Total of mycotoxins	2021	89.8	5951 ^A^	±	6635	7.7	–	44,289	<0.0001 *
	2022	89.9	2861 ^B^	±	2809	0.8	–	15,669	
	2023	75.6	1107 ^C^	±	1253	0.6	–	7225	

^1^ The samples analysed that exceeded the limit of quantification (LOQ) were considered contaminated. The values are expressed as µg/kg adjusted to a moisture content of 12%, ^2^ Maximum content in µg/kg (parts per billion—-ppb) relative to a moisture content of 12%; ^3^ Sum of DON, T-2 and HT-2; ^4^ Sum of DON, ZEN, Fs, T-2 and HT-2; * significant difference *p*-value < 0.05 according to the Kruskal–Wallis Test; ^A–C^ Mean values of the concentrations of mycotoxins detected that differ significantly (*p* < 0.05) according Kruskal–Wallis Test, followed by Dunn’s multiple comparisons test. n.d. Not detected.

**Table 3:** Occurrence of diverse combinations of regulated mycotoxins detected in maize harvested in Serbia during 2021–2023.

Mycotoxins	Proportion of Contaminated Maize Samples (%)		
	2021	2022	2023
AFB_1_ + AFB_2_	9.3	54.8	23.2
AFB_1_ + AFG_1_	1.4	23.8	0.0
AFB_1_ + AFG_2_	0.0	4.8	0.0
AFB_1_ + AFB_2_ + AFG_1_	1.4	23.2	0.0
AFB_1_ + AFB_2_ + AFG_2_	9.3	4.8	0.0
AFB_1_ + AFB_2_ + AFG_1_ + AFG_2_	0.0	4.8	0.0
FB_1_ + FB_2_	81.9	79.2	58.5
Afs * + FB_1_ + FB_2_	18.1	69.6	29.3
Afs * + FB_1_	18.1	72.0	30.5
Afs * + FB_2_	18.1	69.6	29.3
DON + ZEN + FB_1_ + FB_2_	0.0	1.2	1.2
DON + ZEN + FB_1_	0.0	1.2	1.2
DON + ZEN + FB_2_	0.0	1.2	1.2

* At least one of the analysed aflatoxins (B_1_, B_2_, G_1_, and G_2_).

**Table 4:** Occurrence of non-compliance levels of regulated mycotoxins in corn samples harvested in Serbia in 2021–2023 (according to current Serbian and European legislation).

Mycotoxin		Samples Above Compliance Levels for Human Consumption (%) ^1^				Samples Above Compliance Levels for Animal Consumption (%) ^1^		
	ML ^2,3,4^ (µg/kg)	Year			ML ^6^/GL ^7,8,9^ (µg/kg)	Year		
		2021	2022	2023		2021	2022	2023
Aflatoxin B_1_	5 ^2^	8.3	55.4	23.2	20 ^6^/30 ^7^	2.3/0.9	40.5/33.9	12.2/9.8
Sum of aflatoxins	10 ^2^	6.5	50.3	19.5	-	-	-	-
Ochratoxin A	5 ^2^	1.9	20.8	2.4	250 ^8^	0.0	1.8	0.0
Zearalenone	350 ^2^	0.0	1.2	0.6	2000 ^8^/4000 ^6^	0.0	0.0	0.0
Deoxynivalenol	1500 ^3^	0.0	0.0	0.0	8000 ^8^	0.0	0.0	0.0
Sum of fumonisins	4000 ^2,4^	40.1	22.5	1.8	60.000 ^8^	0.0	0.0	0.0
Sum of HT-2 and T-2	100 ^5^	1.4	0.6	0.6	250 ^9^	0.0	0.6	0.0

^1^ Percentages based on the total annual sample sizes (n) of 2021 (n = 216), 2022 (n = 168), and 2023 (n = 164); ^2^ Commission regulation 2023/915/EU [30]; ^3^ Commission regulation 2024/1022/EU [36]; ^4^ Commission Regulation (2024/1756) [37]; ^5^ Commission regulation (2024/1038/EU) [31]; ^6^ Commission directive (2002/32/EC) [32]; ^7^ Serbian Regulation (81/2019) [33]; ^8^ Commission recommendation (2006/576/EC) [34]; ^9^ Commission regulation recommendation (2013/165/EU).

**Table 5:** Method performance characteristics for regulated mycotoxins detected in maize harvested in Serbia during 2021–2023.

Mycotoxin	LOQ (μg/kg)	Apparent Recovery (%)
AFB_1_	0.4	92
AFB_2_	0.4	90
AFG_1_	0.4	86
AFG_2_	0.4	81
OTA	1.6	96
ZEN	16.0	92
DON	64.0	87
FB_1_	40.0	97
FB_2_	40.0	95
HT-2	9.6	93
T-2	9.6	97

## Data Availability

Data are unavailable due to privacy or ethical restrictions. The data presented in this study are not publicly available due to restrictions imposed by the data provider, PATENT CO. Mišićevo. Access to the data may be granted upon reasonable request, subject to obtaining permission from PATENT CO., Mišićevo. Researchers interested in accessing the data are encouraged to contact the authors for further information.

## Supplementary Materials

The following supporting information can be downloaded at: https://www.mdpi.com/article/10.3390/toxins17050227/s1, Table S1. Occurrence of co-contamination with regulated mycotoxins in Serbian maize samples harvested during 2021–2023; the values represent the proportion of maize samples (%) contaminated with multiple mycotoxins. Table S2. *P*-values of Spearman correlation analysis among the levels of regulated mycotoxins contaminating Serbian corn samples harvested during 2021–2023 and their respective localities’ climatic conditions (humidity, rainfall, and temperature). Table S3. *P*-values of Spearman correlation analysis among the levels of regulated mycotoxins contaminating Serbian corn samples harvested during 2021–2023. Figure S1. Heatmap illustration of the Spearman’s correlation coefficients (Rho) among levels of regulated mycotoxins and co-contamination detected in Serbian corn samples harvested during 2021–2023.
