# Supplementary material for: Multi-Mycotoxin Contamination in Serbian Maize During 2021–2023: Climatic Influences and Implications for Food and Feed Safety
Source: Toxins (Basel). 2025 May 4;17(5):227. doi: 10.3390/toxins17050227 (PMC12116126; doi:10.3390/toxins17050227)
Supplement: Supplementary file 1 [file toxins-17-00227-s001.zip › toxins-3461043-supplementary.pdf]

Supplementary Materials

Multi-Mycotoxin Contamination in Serbian Maize During 2021–2023: Climatic Influences and Implications for Food and Feed Safety

Felipe Penagos-Tabares [1,2,*], Anastasija Todorov [3], Jog Raj [4], Hunor Farkaš [4], Goran Grubješić [2], Zdenka Jakovčević [4], Svetlana Ćujić [4], Jelena Nedeljković-Trailović [3] and Marko Vasiljević [4]

[1] CIBAV Research Group, Veterinary Medicine School, Faculty of Agrarian Sciences, Universidad de Antioquia, 050034 Medellín, Colombia
[2] Agromed Austria GmbH, 4550 Kremsmünster, Austria; grubjesic@agromed.at; grubjesic@agromed.at
[3] Department of Animal Nutrition and Botany, Faculty of Veterinary Medicine, University of Belgrade, Bulevar Oslobodjenja 18, 11000 Belgrade, Serbia; tjelena@vet.bg.ac.rs (J.N.-T.)
[4] PATENT CO., 24211 Mišićevo, Serbia; jog.raj@patent-co.com (J.R.); hunor.farkas@patent-co.com (H.F.); zdenka.jakovcevic@patent-co.com (Z.J.); svetlana.cujic@patent-co.com (S.Ć.); marko.vasiljevic@patent-co.com (M.V.)
* Correspondence: felipe.penagos@udea.edu.co

Table S1. Occurrence of co-contamination with regulated mycotoxins in Serbian maize samples harvested during 2021 – 2023, the values represent the proportion of maize samples (%) contaminated with multiple mycotoxins

Group of mycotoxins	Period	Number of mycotoxins per sample								
		0	1	2	3	4	5	6	7	8
Fusarium mycotoxins	3-year period	16.2	7.3	65.5	7.8	3.1	0.2	0.0	0.0	0.0
	2021	11.1	5.5	73.3	7.8	1.8	0.5	0.0	0.0	0.0
	2022	11.2	5.9	70.4	6.5	5.9	0.0	0.0	0.0	0.0
	2023	28.0	11.0	50.0	9.1	0.0	0.0	0.0	0.0	0.0
Aflatoxins	3-year period	59.8	12.4	20.0	6.4	1.5	0.0	0.0	0.0	0.0
	2021	81.1	9.2	7.8	1.8	0.0	0.0	0.0	0.0	0.0
	2022	26.6	17.8	32.5	18.3	4.7	0.0	0.0	0.0	0.0
	2023	65.9	11.0	23.2	0.0	0.0	0.0	0.0	0.0	0.0
Total mycotoxins	3-year period	14.4	7.1	33.5	14.4	14.9	9.8	4.7	1.1	0.2
	2021	10.1	5.5	56.7	15.7	8.3	3.2	0.5	0.0	0.0
	2022	10.1	5.3	10.1	13.6	18.3	23.7	14.8	3.6	0.6
	2023	24.4	11.0	26.8	13.4	20.1	4.3	0.0	0.0	0.0

Table S2. P-values of Spearman correlation analysis among the levels of regulated mycotoxins contaminating Serbian corn samples harvested during 2021 – 2023 and their respective localities' climatic conditions (humidity, rainfall and temperature).

Geo-climatic factors / Mycotoxin	Humidity (Harvest month[1])	Humidity (Growing season[2])	Humidity (Year)	Rainfall (Harvest month[1])	Rainfall (Growing season[2])	Rainfall (Year)	Temperature (Harvest month[1])	Temperature (Growing season[2])	Temperature (Year)	Altitude
Aflatoxin B_1	**>0.0001**	**>0.0001**	**>0.0001**	**>0.0001**	**>0.0001**	**>0.0001**	**>0.0001**	**>0.0001**	**>0.0001**	0.3266
Aflatoxin B_2	**>0.0001**	**>0.0001**	**>0.0001**	**>0.0001**	**>0.0001**	**>0.0001**	**>0.0001**	**>0.0001**	**>0.0001**	0.573
Aflatoxin G_1	**>0.0001**	**>0.0001**	**>0.0001**	**>0.0001**	**0.0005**	**>0.0001**	**>0.0001**	0.0769	0.1801	0.4231
Aflatoxin G_2	**0.0002**	0.9205	0.7371	**0.0001**	**0.0009**	**0.0031**	**0.0006**	0.445	0.5797	0.2329
Ochratoxin A	**>0.0001**	**>0.0001**	**>0.0001**	**>0.0001**	**0.0006**	**>0.0001**	**>0.0001**	**0.0095**	0.1255	0.5176
Zearalenone	0.6011	0.7365	0.2893	0.4686	0.3238	0.3726	0.0572	**0.0014**	**0.0024**	0.5577
Deoxynivalenol	0.9032	0.2639	0.1291	0.0554	0.7132	0.1545	0.4742	0.3471	0.186	0.3958
Fumonisin B_1	0.013	**>0.0001**	**0.0006**	**0.0001**	**>0.0001**	**>0.0001**	**>0.0001**	0.1506	**>0.0001**	**>0.0001**
Fumonisin B_2	0.0452	**>0.0001**	**0.0041**	**>0.0001**	**>0.0001**	**>0.0001**	**>0.0001**	0.2664	**>0.0001**	**>0.0001**
Fumonisins	0.0153	**>0.0001**	**0.0012**	**0.0001**	**>0.0001**	**>0.0001**	**>0.0001**	0.1498	**>0.0001**	**>0.0001**
HT-2	0.1954	0.3158	0.074	0.9899	0.2755	0.4166	0.1306	0.0607	0.3544	0.0597
T-2	0.3692	0.9215	0.2603	0.9354	0.2186	0.8285	0.3628	0.1701	0.8983	**0.0085**
Aflatoxins	**>0.0001**	**>0.0001**	**>0.0001**	**>0.0001**	**>0.0001**	**>0.0001**	**>0.0001**	**>0.0001**	**>0.0001**	0.3005
Trichothecenes	0.932	0.605	0.6148	0.1504	0.7845	0.1561	0.7813	0.9098	0.3895	0.969
Fusarium mycotoxins	**0.0133**	**>0.0001**	**0.0006**	**0.0001**	**>0.0001**	**>0.0001**	**>0.0001**	0.1101	**>0.0001**	**>0.0001**
Total mycotoxins	**0.0043**	**>0.0001**	**0.0005**	**0.0003**	**>0.0001**	**>0.0001**	**>0.0001**	0.1089	**>0.0001**	**>0.0001**
Number of aflatoxins	**>0.0001**	**>0.0001**	**>0.0001**	**>0.0001**	**>0.0001**	**>0.0001**	**>0.0001**	**>0.0001**	**>0.0001**	0.3483
Number of *Fusarium* mycotoxins	0.2067	**>0.0001**	**0.0078**	0.2916	**0.0059**	**>0.0001**	**0.0066**	0.0795	**0.003**	**>0.0001**
Number of mycotoxins	**>0.0001**	**>0.0001**	**>0.0001**	**>0.0001**	**0.0021**	**>0.0001**	**>0.0001**	**>0.0001**	0.2019	**0.0054**

[1] September average; [2] Average of the period (May-September). The Republic Hydrometeorological Service of Serbia provided the geo-climatic data. Values with statistical significance (p-value <0.01) are in bold.

Table S3. P-values of Spearman correlation analysis among the levels of regulated mycotoxins contaminating Serbian corn samples harvested during 2021 – 2023. Values with statistical significance (p-value < 0.05) are in bold.

Parameter \ Parameter	Aflatoxin B_1	Aflatoxin B_2	Aflatoxin G_1	Aflatoxin G_2	Ochratoxin A	Zearalenone	Deoxynivalenol	Fumonisin B_1	Fumonisin B_2	Fumonisins	HT-2	T-2	Aflatoxins	Trichothecenes	*Fusarium* mycotoxins	Total mycotoxins	N° of aflatoxins	N° of *Fusarium* mycotoxins	N° mycotoxins
**Aflatoxin B_1**		**<0.001**	**<0.001**	**<0.001**	**<0.001**	0.521	0.245	**<0.001**	**0.000**	**<0.001**	0.639	0.273	**<0.001**	0.378	**0.000**	**<0.001**	**<0.001**	**<0.001**	**<0.001**
**Aflatoxin B_2**	**<0.001**		**<0.001**	**<0.001**	**<0.001**	0.303	0.081	**0.001**	**0.006**	**0.002**	0.488	0.327	**<0.001**	0.243	**0.004**	**0.000**	**<0.001**	**<0.001**	**<0.001**
**Aflatoxin G_1**	**<0.001**	**<0.001**		**<0.001**	**<0.001**	0.342	0.846	**0.003**	**0.009**	**0.004**	0.413	0.847	**<0.001**	0.481	**0.004**	**0.001**	**<0.001**	**0.001**	**<0.001**
**Aflatoxin G_2**	**<0.001**	**<0.001**	**<0.001**		0.254	0.658	0.767	0.798	0.933	0.828	**0.005**	0.096	**<0.001**	0.065	0.869	0.508	**<0.001**	0.016	**<0.001**
**Ochratoxin A**	**<0.001**	**<0.001**	**<0.001**	0.254		0.148	0.124	**0.002**	**0.008**	**0.003**	0.638	0.166	**<0.001**	0.133	**0.003**	**0.001**	**<0.001**	**0.009**	**<0.001**
**Zearalenone**	0.521	0.303	0.342	0.658	0.148		**0.000**	**0.908**	**0.924**	**0.934**	0.436	0.541	0.529	**0.002**	0.378	0.383	0.674	**<0.001**	**0.004**
**Deoxynivalenol**	0.245	0.081	0.846	0.767	0.124	**0.000**		0.552	0.263	0.499	0.159	0.141	0.266	**<0.001**	0.396	0.492	0.375	**<0.001**	**<0.001**
**Fumonisin B_1**	**<0.001**	**0.001**	**0.003**	0.798	**0.002**	0.908	0.552		**<0.001**	**<0.001**	0.979	0.611	**<0.001**	0.605	**<0.001**	**<0.001**	**<0.001**	**<0.001**	**<0.001**
**Fumonisin B_2**	**0.000**	**0.006**	**0.009**	0.933	**0.008**	0.924	0.263	**<0.001**		**<0.001**	0.609	0.647	**0.001**	0.509	**<0.001**	**<0.001**	**0.000**	**<0.001**	**<0.001**
**Fumonisins**	**<0.001**	**0.002**	**0.004**	0.828	**0.003**	0.934	0.499	**<0.001**	**<0.001**		0.869	0.599	**0.000**	0.615	**<0.001**	**<0.001**	**0.000**	**<0.001**	**<0.001**
**HT-2**	0.639	0.488	0.413	**0.005**	0.638	0.436	0.159	0.979	0.609	0.869		**<0.001**	0.607	**<0.001**	0.532	0.504	0.492	**<0.001**	**<0.001**
**T-2**	0.273	0.327	0.847	0.096	0.166	0.541	0.141	0.611	0.647	0.599	**<0.001**		0.280	**<0.001**	0.982	0.947	0.269	**<0.001**	**0.000**
**Aflatoxins**	**<0.001**	**<0.001**	**<0.001**	**<0.001**	**<0.001**	0.529	0.266	**<0.001**	**0.001**	**0.000**	0.607	0.280		0.418	**0.000**	**<0.001**	**<0.001**	**<0.001**	**<0.001**
**Trichothecenes**	0.378	0.243	0.481	0.065	0.133	**0.002**	**<0.001**	0.605	0.509	0.615	**<0.001**	**<0.001**	0.418		0.323	0.380	0.578	**<0.001**	**<0.001**
***Fusarium* mycotoxins**	**0.000**	**0.004**	**0.004**	0.869	**0.003**	0.378	0.396	**<0.001**	**<0.001**	**<0.001**	0.532	0.982	**0.000**	0.323		**<0.001**	**0.000**	**<0.001**	**<0.001**
**Total mycotoxins**	**<0.001**	**0.000**	**0.001**	0.508	**0.001**	0.383	0.492	**<0.001**	**<0.001**	**<0.001**	0.504	0.947	**<0.001**	0.380	**<0.001**		**<0.001**	**<0.001**	**<0.001**
**N° of aflatoxins**	**<0.001**	**<0.001**	**<0.001**	**<0.001**	**<0.001**	0.674	0.375	**<0.001**	**0.000**	**0.000**	0.492	0.269	**<0.001**	0.578	**0.000**	**<0.001**		**<0.001**	**<0.001**
**N° of *Fusarium* mycotoxins**	**<0.001**	**<0.001**	**0.001**	0.016	**0.009**	**<0.001**	**<0.001**	**<0.001**	**<0.001**	**<0.001**	**<0.001**	**<0.001**	**<0.001**	**<0.001**	**<0.001**	**<0.001**	**<0.001**		**<0.001**
**N° mycotoxins**	**<0.001**	**<0.001**	**<0.001**	**<0.001**	**<0.001**	**0.004**	**<0.001**	**<0.001**	**<0.001**	**<0.001**	**<0.001**	**0.000**	**<0.001**	**<0.001**	**<0.001**	**<0.001**	**<0.001**	**<0.001**	

Values with statistical significance (p-value < 0.01) are in bold.

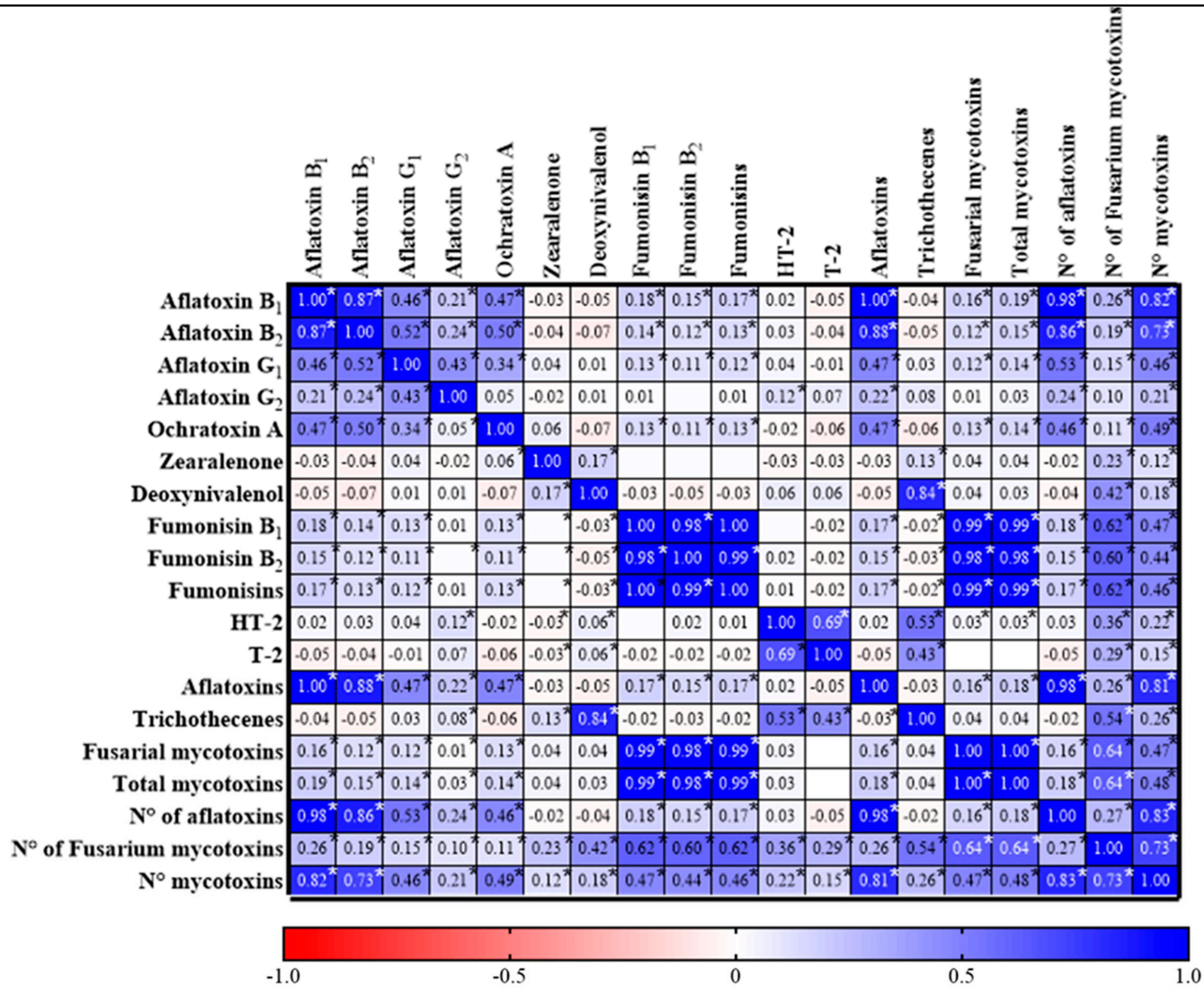

Figure S1. Heatmap illustration of the Spearman's correlation coefficients (Rho) among levels of regulated mycotoxins and co-contamination detected in Serbian corn samples harvested during 2021–2023 The asterisks (*) indicate significant correlation coefficients (p-value < 0.01). All the *p*-values are available in supplementary data Table S3).
